# Metapristone (RU486 metabolite) suppresses NSCLC by targeting EGFR-mediated PI3K/AKT pathway

**DOI:** 10.18632/oncotarget.18640

**Published:** 2017-06-27

**Authors:** Jingwei Shao, Guirong Zheng, Hongning Chen, Jian Liu, Aixiao Xu, Fan Chen, Tao Li, Yusheng Lu, Jianguo Xu, Ning Zheng, Lee Jia

**Affiliations:** ^1^ Cancer Metastasis Alert and Prevention Center, Fujian Provincial Key Laboratory of Cancer Metastasis Chemoprevention and Chemotherapy, Fuzhou University, Fuzhou 350002, China

**Keywords:** non-small cell lung cancer, EGFR, metapristone, cell proliferation, cell apoptosis

## Abstract

Therapies targeting epidermal growth factor receptor (EGFR) can effectively treat with non-small cell lung cancer (NSCLC), but NSCLC’s drug resistance makes it intractable. Herein, we showed that RU486 metabolite metapristone inhibited the proliferation of various NSCLC cell lines with either wild (A549, H1299, H520) or mutated EGFR (H1975, HCC827). The suppression was resulted from inhibition by metapristone of EGFR signaling pathways through down-regulating the EGFR, PTEN, as well as AKT and ERK proteins. In addition, metapristone inhibited anti-apoptotic marker Bcl-2, and activated pro-apoptotic key signaling proteins caspase-3, and poly (ADP-ribose) polymerase. Metapristone induced A549 and H1975 cell cycle via arrest at the G0-G1 stage. What’s more, metapristone inhibited the growth of NSCLC xenografts in BALB/c nude mice through decreasing the expression of tumor growth biomarkers PCNA and EGFR. Taken together, the present study demonstrated that metapristone suppressed NSCLC proliferation by promoting apoptosis via decrease the cellular EGFR-mediated PI3K/AKT pathways. The results suggest metapristone a new treatment for EGFR-overexpressed NSCLC.

## INTRODUCTION

Lung cancer has become the main cause of cancer death in men & women [1, 2]. Lung cancer can be divided into: small cell lung cancer (SCLC) and non-small cell lung cancer (NSCLC), of which NSCLC accounts for 80%∼85% in lung cancer cases [3]. Patients with early stage NSCLC are treated primarily by surgery [4]. Chemotherapy agent is the most common strategy used for NSCLC [5]. But, it is still an agent which needs to improve the outcome of chemotherapy in advanced NSCLC patients. Although molecular targeted agents have been developed in recent years, only a proportion of patients could receive a survival benefit and drug resistance still limited its development. Therefore, it is urgently required to develop a more effective agent for NSCLC therapy.

EGFR is the earliest identified driver oncogenes for development of lung tumors. EGFR is overexpressed in about 50% of NSCLC tumors and correlates with poor prognosis [6]. EGFR targeted therapies have been developed in preclinical studies as well as clinical use in patients [7], the most successful targeted therapeutic drugs are monoclonal antibodies, that directly interfere with ligand-receptor binding and EGFR tyrosine kinase inhibitors (osimertinib and erlotinib *etc.*), which are effectively used in patients with advanced EGFR-mutated NSCLC [8]. Patients inevitably develop primary resistance and secondary resistance to EGFR-targeted therapies, which is in part due to K-ras gene mutation [9], PTEN deletion [10], transformation of epithelial cells (EMT) [11], MET amplification [12] and hepatocyte growth factor (HGF) overexpression [13], *etc*. Resistance to EGFR inhibitors can also arise through persistence or reactivation of ERK1/2 signaling [14]. However, only a small series of the resistance mechanisms have been identified [15, 16]. Hence, efforts are ongoing for the development of multi-targeted therapeutic agent that blocks RTK activities and downstream signaling of EGFR to improve therapeutic efficacy and overcome drug resistance.

RU486 (mifepristone) has the activity of anti-progesterone and anti-glucocorticoid [17]. It is widely applied for anti-pregnancy [18] and hormone-dependent tumors, e.g. breast cancer [19], endometrium cancer [20], prostate cancer [21] and ovary cancer [22]. Mifepristone can also inhibit different cancer cell growth regardless of the expression of hormone responsiveness. Recently, wang *et al* reported that glucocorticoids might compromise the effect of EGFR-TKIs gefitinib in NSCLCs [23]. Given the anti-glucocorticoid activity of metapristone is similar to mifepristone [24–28], we hypothesized that metapristone could effectively inhibit the growth of NSCLC by synergizing with gefitinib. Very recently, we reported that metapristone could inhibit the migration of NSCLC cells through suppressing RAS/RAF/MEK/MAPK signaling pathways [29]. On the basis of our results, we believe that metapristone has the potential to inhibit the growth of NSCLC, and may be a good candidate for overcoming resistance to chemotherapy. Herein, we investigated the pro-apoptotic activities of metapristone in different kinds of NSCLC cells *in vitro* as well as the inhibition effect of metapristone on the growth of NSCLC xenografts *in vivo*. We further explored the potential molecular mechanisms of metapristone, including the differentially expressed of proteins and intracellular signal pathways in NSCLC cells after metapristone treatment, and especially investigated the effects of metapristone on inherent and growth factor-induced phosphorylation of EGFR. Our study showed that metapristone could be developed as a potent inhibitor of EGFR in NSCLC cells.

## RESULTS

### Screening of erlotinib-resistant cell lines

The sensitivity to erlotinib growth-inhibitory effects were evaluated in a panel of five NSCLC (H1299, A549, H520, H1975, and HCC827) cell lines [30, 31] (Table 1). Cells were treated with erlotinib (0∼50 μM) for 48 h. As shown in Table 1, among different NSCLC cell lines, HCC827 cell was the most sensitive to erlotinib. We could also find that metapristone (0∼20 μM) could effectively inhibit the proliferation of different NSCLC cells. There was no direct correlation between gene mutation pattern and sensitivity to metapristone. Among the NSCLC cell lines, H1975 and A549 cells were more sensitive to metapristone and were selected for the following studies.

**Table 1:** Screening for erlotinib-resistant/ sensitive cells

NSCLC cells	Gene mutation				Erlotinib (μM)	Metapristone (μM)
	EGFR	KRAS	NRAS	BRAF		
A549	WT	Mut (G12S)	WT	WT	36.59±2.47	19.28±1.68
H1975	Mut (T790M) Mut (L858R)	WT	WT	WT	29.00±2.78	17.64±2.79
H1299	WT	WT	Mut (Q61K)	WT	35.07±3.12	21.59±1.82
H520	WT	Mut	WT	WT	27.59±2.49	21.67±2.56
HCC827	Mut(E746-A750)	WT	WT	WT	0.17±1.78	24.27±2.86

Erlotinib IC_50_>10 μM: erlotinib-resistant, Erlotinib IC_50_<10 μM: erlotinib-sensitive, WT: wild type, Mut: mutated, Del: deletion.

### Effects of metapristone on cell viability

**Figure 1:**
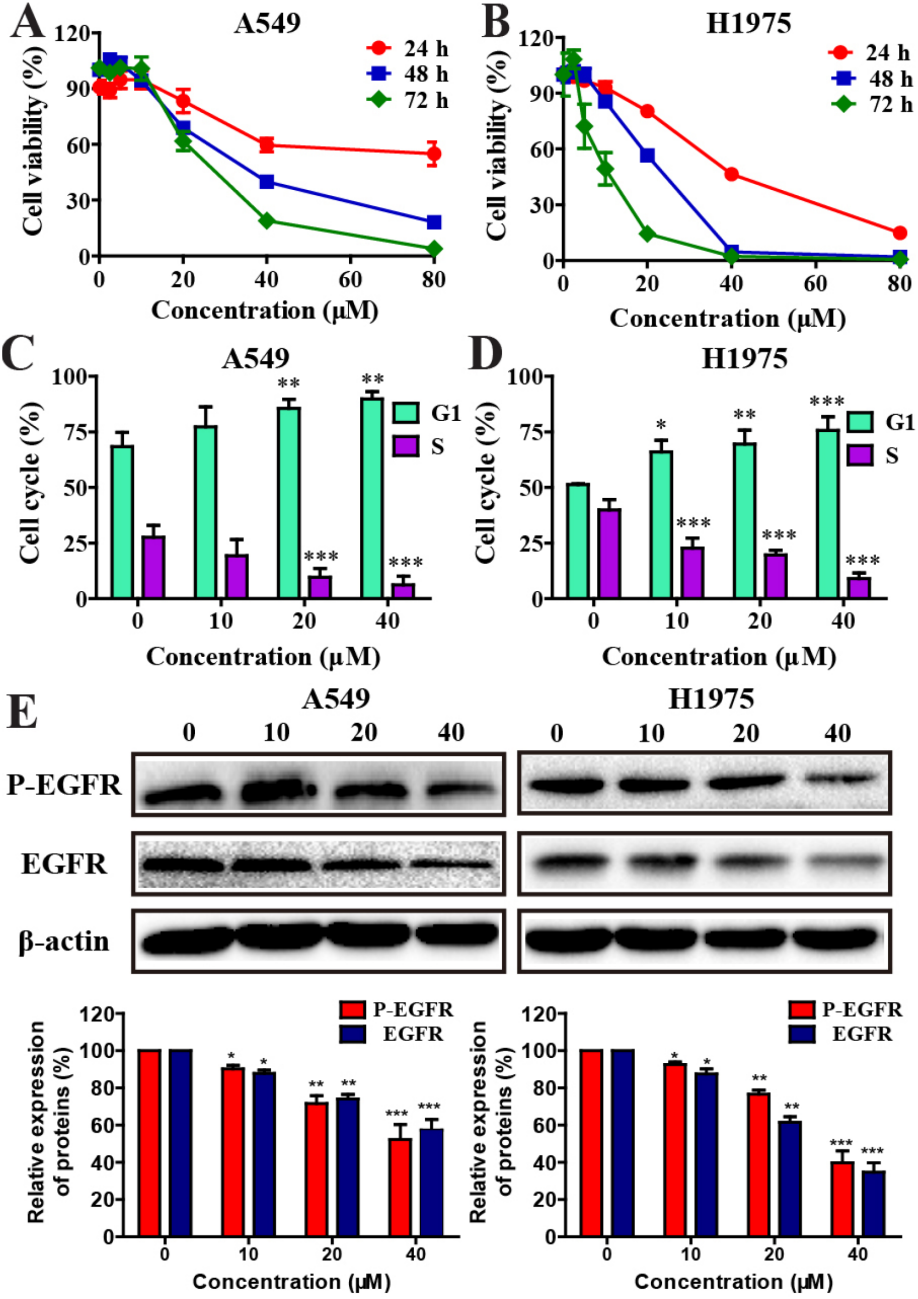
Effects of metapristone on non-small cell lung cancer cell viability **(A and B)** Cells were incubated with different concentration of metapristone for 24, 48 or 72 h. Flow cytometry analyzed the cell cycle distribution in A549 **(C)** and H1975 **(D)** cells. **(E)** Western blotting (upper) and related quantitative analysis (lower) showed the changes of P-EGFR and EGFR in A549/H1975 cells induced by metapristone treatment. Date were the mean ± SEM (n=3). * P<0.05, ** P<0.01 and *** P<0.001, compared with the control.

NSCLC cells were treated with metapristone for 24 h up to 72 h to determine whether metapristone could inhibit the growth of NSCLC cells. The results showed that metapristone could effectively inhibit A549 and H1975 cells proliferation in a time-dependent and concentration-dependent manner (Figure 1A and 1B). The IC_50_ values for metapristone to suppress A549 and H1975 cells were estimated to be 80.55 ± 12.13 μM and 33.29 ± 3.69 μM for 24 h, respectively, indicating that H1975 cells are more sensitive to metapristone compared with that of A549 cells.

### Effects of metapristone on cell cycle distribution

The effect of metapristone on A549 and H1975 cell cycle distributions were examined. The results showed that NSCLC cells treated with metapristone for 24 h caused an increased accumulation of cells at the G0/G1 phase and a corresponding decrease at S phase (Figure 1C and 1D). Furthermore, the expression levels of EGFR and P-EGFR proteins related to cell cycle were examined. As shown in Figure 1E, metapristone could effectively decrease the expression of EGFR and P-EGFR in A549 and H1975 cells.

### Metapristone induces apoptosis in A549 and H1975 cells

**Figure 2:**
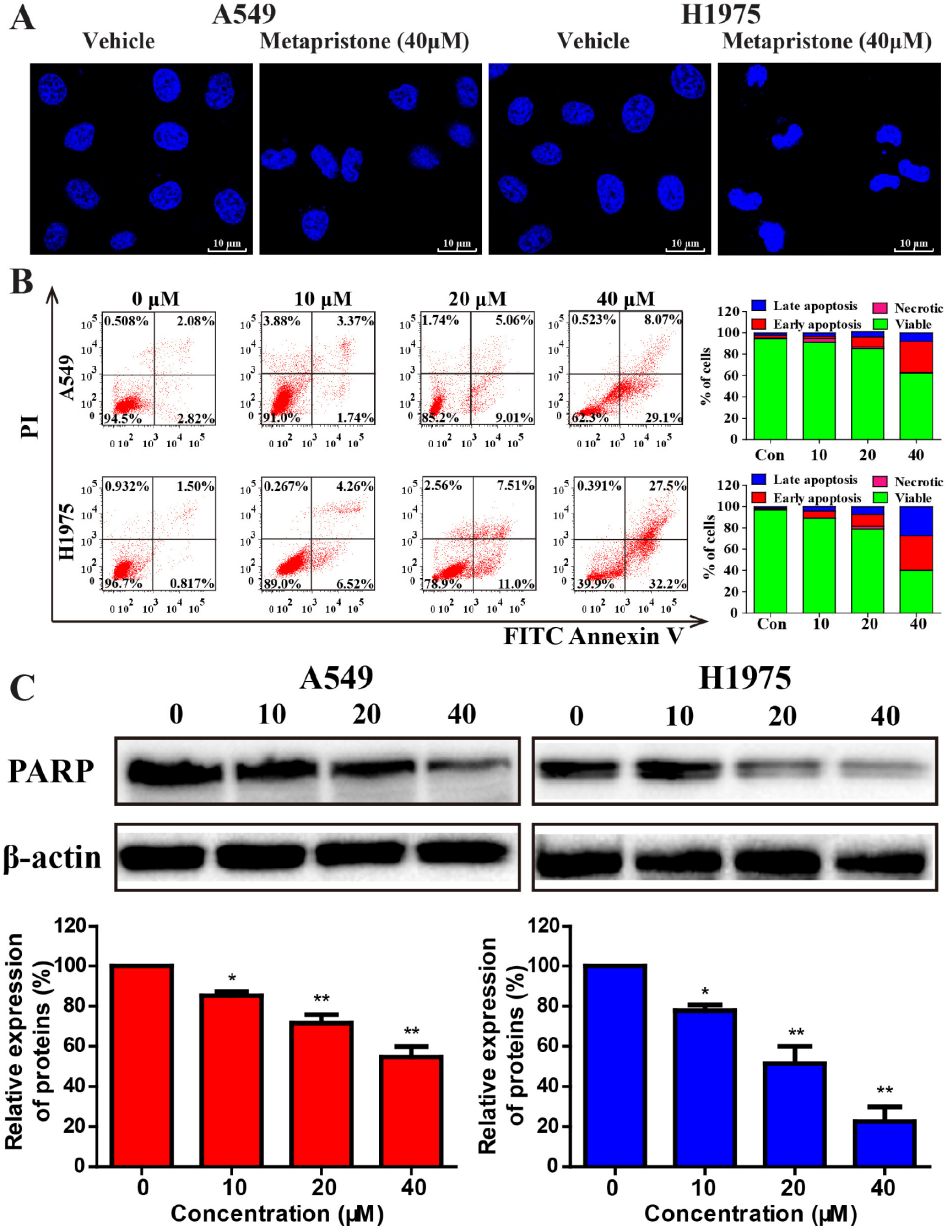
Effects of metapristone on non-small lung cancer apoptosis in A549 and H1975 cells **(A)** A549 and H1975 cell morphological changes and nuclear alterations after metapristone treatment. The condensed and fragmented fluorescent nuclei of apoptotic cells are visible in the treated cells, but not in the control cells. **(B)** Flow cytometric analyzed cell apoptosis in A549 and H1975 cells using the Annexin V-FITC/PI dual-labeling technique. Quantified values were shown on the right. **(C)** The levels of the typical apoptosis-related proteins total PARP were determined by Western blotting (upper). The expression of total PARP was quantified by using Image Lab analysis software. The condensed and fragmented fluorescent nuclel (white arrows) of apoptotic cells are visible in the treated cells, but not in the control cells. Date were the mean ± SEM (n=3). * P<0.05, ** P<0.01 and *** P<0.001, compared with the control.

The induction of apoptosis, DAPI staining and western blot were used to determine whether metapristone could inhibit the growth of tumor cells. A549 and H1975cells exhibited a typical morphological feature of apoptosis (cell shrinkage) after treatment of metapristone for 24 h. Cell apoptosis were further confirmed by DAPI staining. A549 and H1975 cells treated with metapristone (40 μM) displayed bright-blue fluorescent condensed nuclei and nuclear shrinkage, whereas control cells showed round and homogeneous nuclei (Figure 2A). Next, the apoptotic effect of metapristone in A549 and H1975 cells were determined by flow cytometry. As shown in Figure 2B, compared with control group (4.8 % cells apoptotic), A549 cells treated with 40 μM metapristone for 24 h caused 37.17 % cells apoptotic and observed 57.9 % cells apoptotic in H1975 cell lines (Figure 2B). Western blot assay showed that metapristone treatment resulted in a decrease of PARP protein, which was related to the later stages of apoptosis (Figure 2C).

### Effects of metapristone on cell apoptosis via caspase-3 dependent pathway

**Figure 3:**
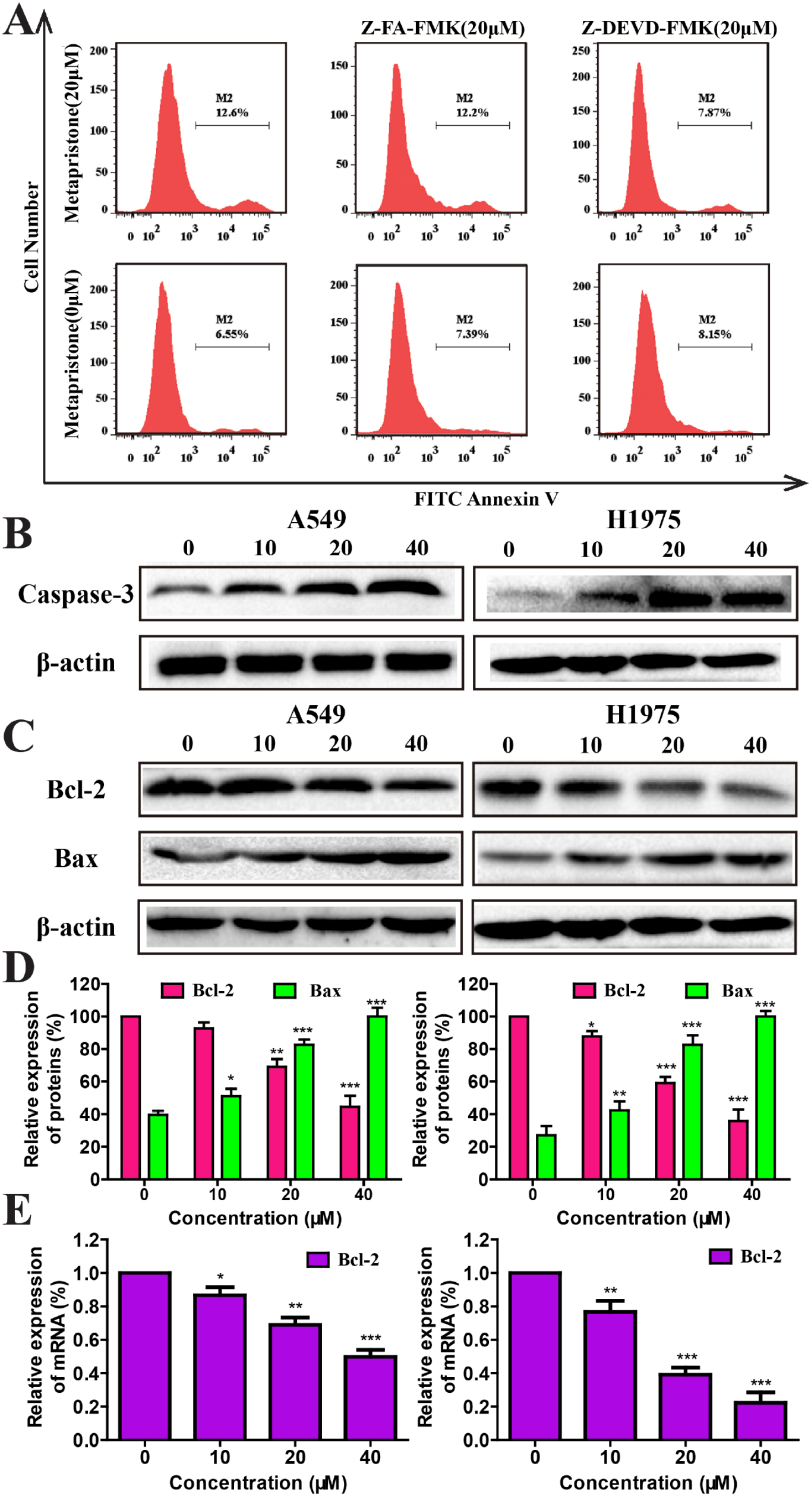
Mechanism of metapristone induced apoptosis on non-small lung cancer **(A)** Detection of caspase-3 activity by flow cytometric analysis of apoptosis in A549 cells. A549 cells were pre-incubated with the following: no inhibitor, 20 μM of a negative control inhibitor Z-FA-FMK or 20 μM of caspase-3 inhibitor Z-DEVD-FMK for 30 min, and then either left untreated (bottom row) or treated with 40 μM of metapristone for 24 h (top row). **(B and C)** The expression caspase-3(B), Bcl-2 and Bax **(C and D)** were determined by western blotting in A549 and H1975 cells after metapristone treatment. The quantitative analysis of the expressions of Bcl-2 in A549 or H1975 cells under different concentrations of metapristone were detected by RT-PCR assay. **(E)** Each bar represents the mean ± SEM (n=3). * P<0.05, ** P<0.01 and *** P<0.001, compared with the control.

To further determine the potential mechanism of metapristone on the apoptotic process, we evaluated the expression of some apoptosis associated proteins, including Bax and caspase-3 as well as apoptosis-related protein Bcl-2 by flow cytometry, western blot and qRT-PCR assay. Flow cytometry assay revealed that after cells treated with caspase-3 inhibitor Z-DEVD-FMK could effectively decrease the extent of cellular apoptosis (Figure 3A). Western blotting results showed that metapristone could effectively increase the levels of caspase-3 in A549 and H1975 cells (Figure 3B) indicated that metapristone might via the mitochondrial pathway induced apoptosis [32]. We also investigated the effects of metapristone on apoptosis-related protein of Bcl-2 and Bax. As shown in Figure 3C and 3D, metapristone could effectively decrease the expression of Bcl-2 proteins and increase expression of Bax proteins in both cell lines. In qRT-PCR assay (Figure 3E), metapristone dose-dependently inhibited the mRNA expression of Bcl-2. These data demonstrated that the metapristone-induced apoptosis was dependent on the caspase-3dependent pathway.

### Metapristone suppressed the expression of pivotal proteins in the PI3K/AKT and ERKs pathways in A549 and H1975 cells

**Figure 4:**
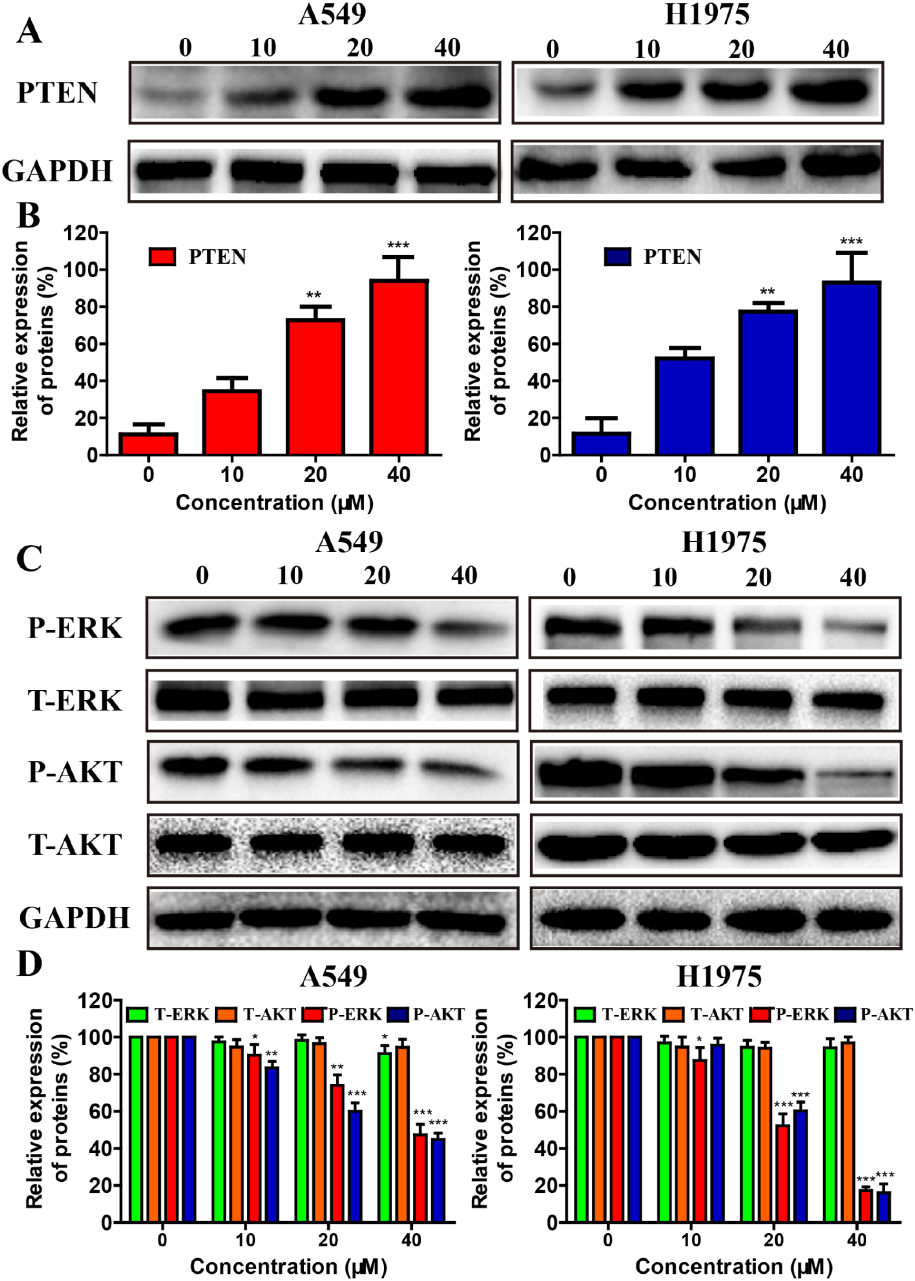
Effects of metapristone treatment on PTEN/AKT, and ERKs signal pathway in NSCLC cells **(A)** A549 and H1975 cells treated with 10-40 μM metapristone for 24 h was to determine its effect on the expression PTEN proteins. **(B)** Related quantitative analysis showed changes PTEN in A549 and H1975 cells induced by metapristone treatment. **(C and D)** Western blotting **(C)** and related quantitative **(D)** analysis showed changes in expression of P-AKT, T-AKT, P-ERK and T-ERK in A549 cells, and of P-AKT, T-AKT, P-ERK and T-ERK in H1975 cells induced by metapristone. The date expressed as the mean ± SEM (n=3). * P<0.05, ** P<0.01 and *** P<0.001, compared with the control.

Resistance to EGFR inhibitors may be driven by many different mechanisms including the activation of PI3K/AKT and MEK/MAPK signaling pathways [33], both of which play an important roles in the tumorigenesis, cell proliferation, angiogenesis, and cell apoptosis [34].PTEN, AKT, ERK proteins levels were also evaluated after metapristone treatment for 24 h. As shown in Figure 4A-4D, metapristone clearly increased the expressive levels of PTEN in A549 and H1975 cells. Compared with control group, the expressive levels of P-AKT and P-ERK proteins in the metapristone treatment groups were much lower but the expressive levels of T-AKT and T-ERK proteins in the metapristone treatment groups didn’t show obvious changes.

### Metapristone inhibits the P-EGFR in A549 and H1975 cells

**Figure 5:**
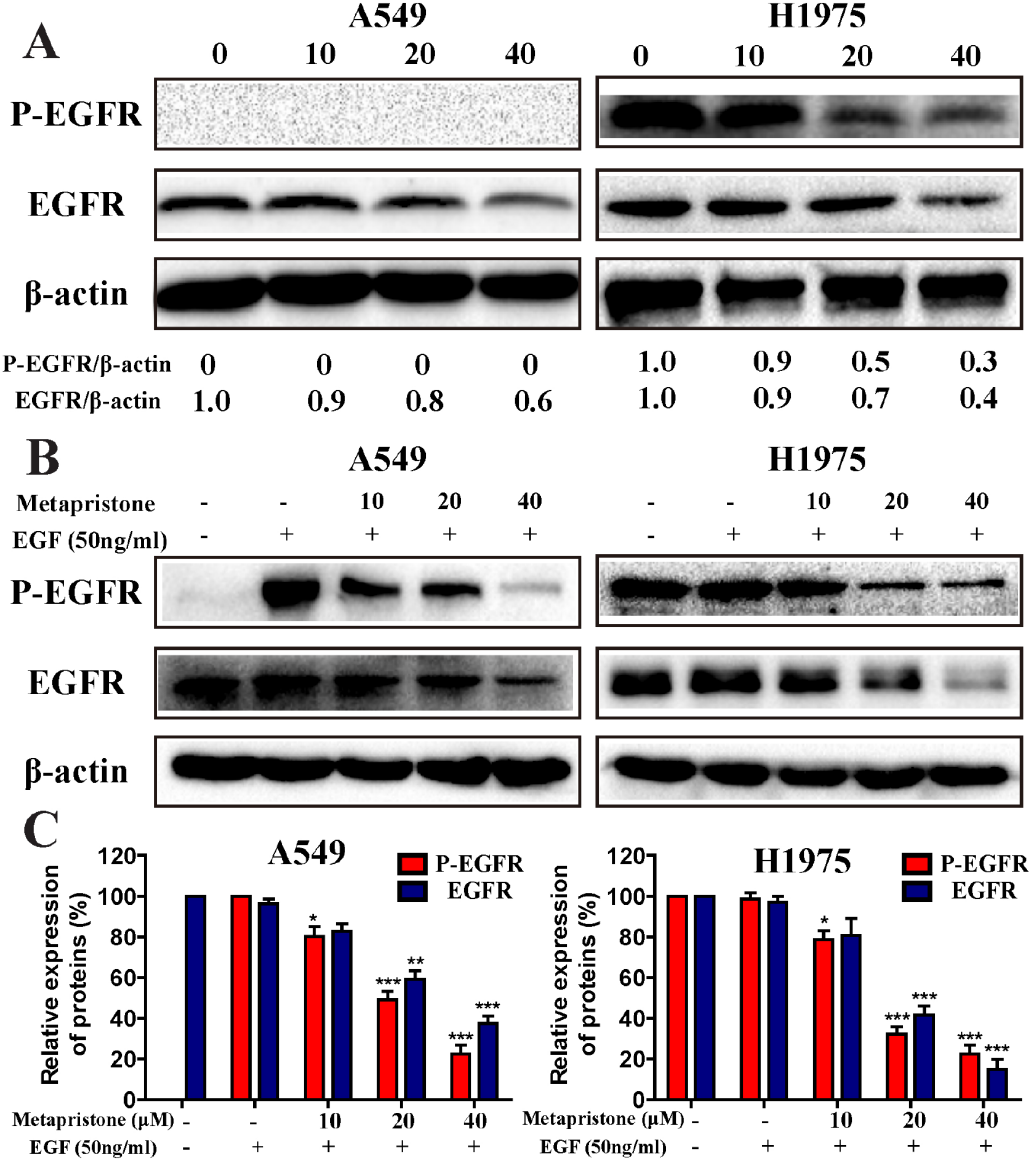
Effect of metapristone treatment on the inherent and EGF-induced phosphorylation of EGFR in A549 and H1975 cells (A) A549 and H1975 cells were treated with metapristone (10, 20, 40 μM) for 24 h in serum-free cell medium. (B) Serum starved A549 and H1975 cells were treated with metapristone (10, 20, 40 μM) for 24 h and then incubated without or with EGF (50 ng/mL) for 30 min. (C) Related quantitative analysis showed changes in EGFR and phosphorylated-EGFR of A549 and H1975 induced by EGF treatment. Each bar represents the mean ± SEM (n=3). * P<0.05, ** P<0.01 and *** P<0.001, compared with the control.

EGFR and its downstream signaling pathways are closely related to cell proliferation, cancer progression, metastasis and angiogenesis [35]. To determine whether metapristone could effect on EGFR expression, EGFR over-expressing A549 and H1975 cells were treated with metapristone (10, 20, 40 μM; 24 h). The result showed that metapristone could effectively reduce the expression of EGFR, but the expressive levels of phosphorylated EGFR (P-EGFR) proteins in the metapristone treatment groups didn’t show obvious changes in A549 cell (Figure 5A). However, metapristone dose-dependently inhibited the expression levels of EGFR and P-EGFR in H1975 cells. We further determined whether metapristone could affect the expression on P-EGFR. We found that can be effective in treating extraordinary inhibited P-EGFR (Figure 5B and 5C). In H1975 cells, we also can find the similar effect of metapristone on P-EGFR. These consequences indicate that metapristone decreases both inherent and EGF-induced phosphorylation of EGFR.

### Metapristone inhibits human NSCLC xenografts in nude mice

**Figure 6:**
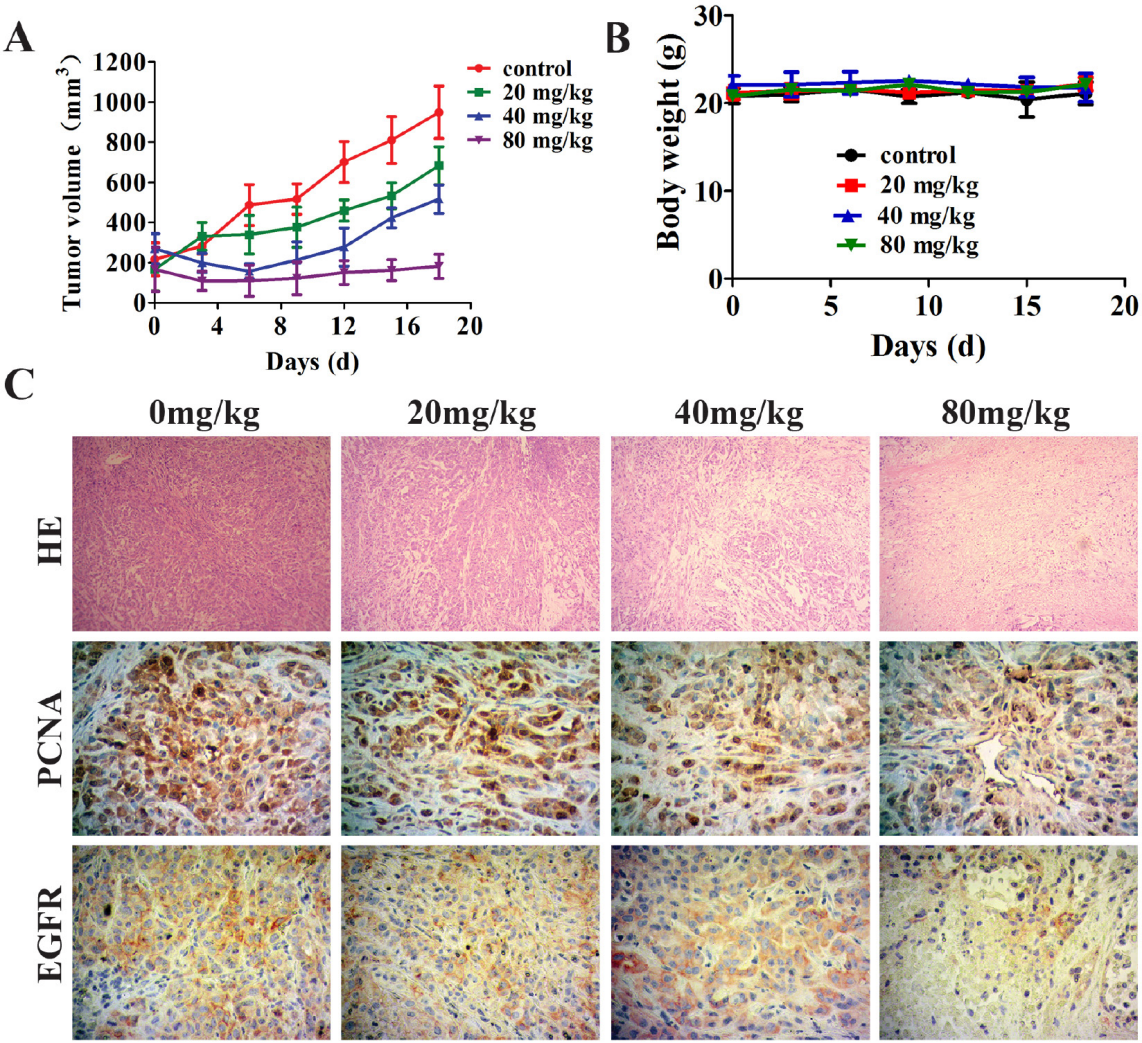
Effect of metapristone in non-small lung cancer xenograft model A549 cells were injected subcutaneously to the athymic nude mice. Two week after the injection, metapristone (20 mg/kg/day) was given to the mice for 18 days (n = 7) (A) Non-small lung cancer tumor volume results after treatment with metapristone. (B) Body weight change during the treatment periods. (C) Hematoxylin and Eosin (H&E) staining of paraffin-embedded non-small cell lung cancer tumors; Cell proliferation was detected by PCNA staining. Cell survival signaling was detected by EGFR staining. PCNA or EGFR-positive tumor cells are brown and the arrows indicate PCNA or EGFR-positive tumor cells.

To further investigate the anti-tumor activity of metapristone, we examined the anti-tumor efficacy of metapristone with a human NSCLC tumor xenografts model in nude mice. The results showed that the tumor volume decreased more rapidly with the dose of metapristone increasing (Figure 6A). However, when the administrated dose of metapristone in mice increased, the body weights of mice didn’t show obvious changes (Figure 6B). These data proved that metapristone could effectively anti-tumor growth *in vivo*. Moreover, H&E staining revealed that the metapristone treated tumors were less aggressive and necrotic than the control tumors (Figure 6C). The expression of cell proliferation marker protein PCNA was examined by immunohistochemical (IHC) staining (Figure 6C). The results showed that metapristone treatment groups had a lower expression of PCNA compared with the untreated control group. This was accompanied by a decreased level of EGFR, which are critical in survival signaling pathways (Figure 6C). Overall, these IHC results correlate well with the antitumor capacity of metapristone shown in the xenograft studies.

## DISCUSSION

NSCLC has become the main cause of cancer death in men & women. Targeted therapy against EGFR is now widely used in clinic to treat NSCLC patients. Cross *et al* [36] and Tang *et al* [37] reported that a newest FDA-approved epidermal growth factor receptor (EGFR) tyrosine kinase inhibitor osimertinib could be used for non-small cell lung cancer (NSCLC) patients with EGFR T790M mutation. It could effectively target both sensitizing and resistant T790M (+) mutant EGFR. Herein, we sought to assess the anti-proliferative, pro-apoptotic and anti-tumor activities of metapristone, a predominant metabolite of miferistone, against NSCLC cells with wild type EGFR (A549) or mutant type EGFR (H1975) both *in vitro* and *in vivo*.

Metapristone could effectively inhibit the growth of breast cancer cells, colorectal cancer and B_16_F_10_ melanoma carcinomas [26, 38]. Moreover, metapristone may exert its chemopreventive activities through intervened the EMT-related signaling pathways in breast cancer cells [27]. In this study, consistent with previous findings, we demonstrated that metapristone could effectively inhibite the cell growth in NSCLC cells (Figure 1A and 1B). Besides, we observed that metapristone arrested A549 and H1975 cells at the G0/G1 stage (Figure 1C and 1D). Moreover, western blot assay results showed that metapristone significantly degraded the EGFR and P-EGFR protein expression in A549 and H1975 cells (Figure 1E). In addition, by morphology study, DAPI staining and Annexin V/PI double staining, we showed that metapristone could induce apoptosis in A549 and H1975 cell lines (Figure 2A, 2B and 2C). These results raised the possibility that metapristone exerted an anticancer action in the EGFR mutant and EGFR wild-type NSCLC cell lines. In order to understand the molecular mechanisms of action, we further examined the protein expressions related to the proliferation, cell cycle, apoptosis and suppressor gene in the A549 and H1975 cells.

Activation of apoptosis is regarded as a good target for cancer therapy [39]. Apoptosis is affected by anti-apoptotic and pro-apoptotic proteins of the Bcl-2 family, and is executed via caspase. Caspase-3 is a critical protein of apoptosis, which can effectively affect the pathogenesis and treatment of a variety of malignancies [40]. The expression levels of PARP were significantly decreased after metapristone treatment (Figure 2C). We also found that metapristone could effectively increase the expression of caspase-3 in A549 and H1975 cells (Figure 3A and 3B). Induction of apoptosis was usually accompanied by decreasing the expression of Bcl-2 proteins/genes and increasing the expression of Bax proteins (Figure 3C, 3D and 3E). Taken together, these results showed that metapristone significantly inhibited the growth of NSCLC cells and remarkably induced the apoptosis by activating caspase-3 pathways, enhancing PTEN protein levels, and reducing PARP and PCNA protein levels, as well as decreasing the protein level of CyclinD1.

Activation and overexpression of EGFR play a significant role in NSCLC cells. Metapristone could effectively reduce the constitutive and P-EGFR proteins expression in NSCLC cells (Figure 5A, 5B and 5C). We also investigated the effects of metapristone on PI3K/ AKT and ERKs pathways to confirm the blocking effects of metapristone on EGFR downstream signaling pathways. Activation of PI3K/AKT and ERKs pathways could lead to cell apoptosis. AKT is a cytosolic signal transduction protein kinase that plays a crucial role in tumor progression by inhibiting apoptosis [41]. PI3K/AKT pathways play an important role in the induction of AKT activity. MAPK/ERK pathways can link extracellular signals to the machinery that controls fundamental cellular processes [42]. Suppression of AKT and the ERK pathway by antiprogesterone/antiglucocorticoid agents inhibited the migration and growth as well as induced the apoptosis in human breast and ovarian cancer cells [43, 44]. Herein, we found that metapristone significantly degraded the expressions of phosphorylated AKT and ERK proteins in A549 and H1975 cells (Figure 4D). Furthermore, we also performed the *in vivo* tumor growth assay to determine the relevance of these *in vitro* findings. The *in vivo* result showed that metapristone could significantly inhibit A549 tumor growth in BALB/C nude mice (Figure 6).

In summary, the present study identifies metapristone as an effective inhibitor of EGFR in NSCLC cells. Our finding suggested that metapristone could effectively inhibit the proliferation and apoptosis of NSCLC cells. Metapristone could induce significant cell apoptosis via targeting EGFR and its downstream PTEN/AKT and ERKs signaling pathways by up-regulating the level of PTEN, and down-regulating the expression of P-AKT and P-ERK proteins, as well as activating the apoptotic-related proteins caspase-3 in NSCLC cells. Overall, these observations provide evidence that metapristone possesses anti-tumor activity in NSCLC cells both *in vitro* and *in vivo*. Our present study indicated that metapristone could be an useful agent for the treatment of NSCLCs.

## MATERIALS AND METHODS

### Materials and reagents

Mifepristone (RU486, purity >98%) [RU486, 17β-hydroxy-11β-(4-dimethylamino) phenyl-17α-(1-propynyl)-estra-4, 9-dien-3-one], MW 429.59, was purchased from Shanghai New hualian pharmaceutical Co., China. Compound metapristone [N-monodemethyl RU486, (or 17β-hydroxy-11β-(4-dimethylamino) phenyl-17α-(1-propynyl)-estra-4, 9-dien-3-one), MW 415.57] was synthesized as previously reported [38]. Erlotinib was purchased from Aladdin Industrial Corporation (Shanghai, China). Other reagents were obtained from commercial suppliers in analytically pure or chemically pure forms. The monoclonal and polyclonal antibodies for EGFR, phosphor-EGFR (Tyr 1068) and PTEN were purchased from Cell Signaling Technology (Beverly, MA). The antibodies for phosphor-ERK1/2 (phospho-p44/42, Thr202/Tyr204), phosphor-AKT, Bcl-2, CyclinD1, PARP and caspase-3 were purchased from Santa Cruz Biotechnology, Inc. (Santa Cruz, CA). The polyclonal antibodie for PCNA was purchased from Abcam. The secondary antibody goat anti-mouse-IgG horseradish peroxidase was obtained from Dingguo Changsheng Biotechnology Co., Ltd, China.

### Cell lines and cell culture

Human non-small cell lung cancer A549, H1975 and H1299 cell lines were purchased from the Cell Bank of the Chinese Academy of Sciences (Shanghai, China). Human non-small lung cancer H520 and HCC827 cells were a gift from Kai Wang (Harbin Medical University). A549 cell was cultured in F-12K medium; H1975, H1299, H520 and HCC827 cells were cultured in RPMI-1640 medium supplemented with heat-inactivated fetal bovine serum (FBS, 10%), penicillin (100 U/mL), and streptomycin (100 μg/mL) in a humidified atmosphere of 5% CO_2_ at 37 °C. Metapristone was dissolved in dimethyl sulfoxide (DMSO) and was used in all experiments. The final concentration of DMSO used was 0.1% (v/v) for each treatment. For dose-dependent studies, A549 and H1975 cells were treated with metapristone (10-40 μM) in F12K or RPMI 1640 mediums containing 2% FBS for 24 h. Control cells were treated with the vehicle alone. In additional experiments, serum starved A549 and H1975 cells were treated with metapristone (10-40 μM; 24 h and then incubated with or without EGF (50 ng/mL; 30 min).

### Cell viability

The cytostatic effect of metapristone was determined by the 3-[4, 5- dimethylthiazol]-2, 5-diphenyltetrazolium bromide (MTT) assay as previously described [45]. Cells (A549 and H1975) were plated in 96-well plates and treated with 5-80 μM concentrations of metapristone for 24 h, 48 h and 72 h, respectively. After incubation for specified times at 37 °C in a humidified chamber, MTT reagent (5 mg/mL in PBS) was added to each well and incubated for another 4 h. The MTT solution was removed from the wells by aspiration and the formazan crystals were dissolved in DMSO (150 μL). Absorbance was detected on a microplate ELISA reader (Tecan, Switzerland) at 570 nm. The concentration of metapristone which gives a 50% growth inhibition value was defined as the IC_50_.

### Cell cycle

Cell cycle distribution was analyzed by flow cytometry as previously described [46–48]. Briefly, A549 and H1975 cells were treated with metapristone for 24 h, digested with trypsin, collected by centrifugation at 1500 rpm for 5 min, and washed twice with ice-cold PBS. The cell pellet was washed three times with PBS and fixed in 70% ethanol overnight at 4°C. The concentrated samples were incubated with fluorescent solution (1% (v/v) Triton X-100, 0.01% RNase, 0.05% PI) for 30 min at 37 °C staining in darkness. The cell cycle distribution was then measured by flow cytometry. Data were processed and analyzed by using Modfit software. All experiments were performed three times.

### Apoptosis assessment by DAPI staining

A549 and H1975 cells were seeded in 24-well plates at a density of 5×10^4^ cells/well and incubated overnight. Cells were treated with metapristone (40 μM) for 24 h. After treatment, cells were washed twice with PBS, then stained with 10 μg/mL DAPI (Sigma, St. Louis, MO) in PBS at 37 °Cfor 10 min in the dark. Apoptotic cells were detected with a fluorescence microscope (ZEISS), and data were collected from three independent experiments.

### Apoptosis analysis by flow cytometry

Annexin V-FITC/PI double staining kit was similar to that described previously [29]. A549 and H1975 cells were treated with different concentrations (0, 10, 20, 40 μM) of metapristone for 24 h. After treatment, the cells were harvested by centrifugation at 1500 rpm for 5 min, then suspended with 500 μL of 1 X binding buffer and stained with 10 μL of the solution containing Annexin V-FITC (5 μL) and PI (5 μL) for 15 min in the dark according to the manufacture of instructions. Cells were then analyzed by using flow cytometry (BD Bioscience, FACS Aria III). All experiments were performed three times.

### Caspase-3 activity assay

A549 and H1975 cells were pre-incubated with caspase-3 inhibitor Z-DEVD-FMK (20 μM) or negative control inhibitor Z-FA-FMK (20 μM) for 30 minutes at 37 °C. After A549 and H1975 cell lines were treated with 40 μM of metapristone for 24 h, cells were collected and stained with FITC Annexin V to identify cells undergoing apoptosis. Then the cells were analyzed by flow cytometry (BD FACS Aria III). All experiments were performed three times.

### Western blot analysis

Western blotting was performed as described previously [29]. Briefly, A549 or H1975 cells were seeded in 6-well plates at density of 5×10^5^ cells/well and incubated overnight. Then, the cells were treated with various concentrations of metapristone (0, 10, 20, 40 μM) for 24 h. Cells were suspended in ice-cold lysis buffer and lysed for several seconds, the lysates were clarified by centrifugation at 12,000 g for 5 min under 4 °C. Protein concentrations were measured by BCA assay. Samples with equivalent amounts of proteins were resolved by SDS-PAGE using a 10% gel. Proteins were transferred to a polyvinylidene fluoride (PVDF) membrane. The membrane was blocked with 5% skim milk in Tris-buffer saline containing 0.1% Tween 20 (TBST) and then incubated with specific primary polyclonal or monoclonal antibodies for overnight at 4 °C. After washing in TBST, the membranes were incubated with goat anti-rabbit (1:10000) or anti-mouse (1:10000) IgG HRP-labeled secondary antibodies for 1 h at room temperature. After washing with TBST and TBS, the particular protein was visualized with the ECL kit. Protein band were quantified with Image software and normalized by GAPDH or β-actin bands for analysis. Each experiment was done in triplicate.

### Quantitativereal-time PCR

RT-PCR assay as described previously [49]. Briefly, total RNA were isolated from A549 and H1975 cells using TRIzol reagent (Invitrogen). The RNA was quantified at OD_260/280_ and 1 μg RNA was used for reverse transcription using the Prime Script® RT reagent Kit (TaKara, Dalian, China) according to the manufacturer’s instructions. Amplification of specific PCR products were carried out using the SYBR® Premix Ex Taq™ PCR kit (Takara, Dalian, China). The designed primers in this study were: β-actin forward primer, 5′-TGGCACCCAGCACAATGAA-3′; β-actin reverse primer, 5′-CATAGTCATAGTCCGCCTAGAAGCA-3′. The relative gene expression for each sample was calculated by the 2^-ΔΔCt^ method, which reflected the target mRNA expression normalized to β-actin mRNA levels. Each value represents the mean from triplicate trials ± SD.

### *In vivo* tumor growth assay

A549 cells were harvested by centrifugation, washed with PBS and re-suspended to get the suitable in serum-free F12K medium. Then, cells (5×10^6^/100 μL) were injected subcutaneously behind the right shoulder of nude mice. The tumors were allowed to grow until they reached a volume of 100 mm^3^. The animals were then randomly assigned to four groups. (n = 8 for each group), and then they were orally gavaged with either soybean oil (control) or metapristone (20, 40 and 80 mg/kg) for 18 days, respectively. Tumor volume and animal weight were measured every three days for 7 times. Tumor volumes were calculated from digital caliper measurements (volume = 1/2 × (L× W^2^); L= longer diameter and W = shorter diameter). After 186 days of treatment, the tumors were harvested and wet weights were determined. The tumor were excised, washed with PBS, and fixed in 10% neutral buffered formalin, and then paraffin embedded and stained with hematoxylin and eosin (H&E). The histological observations were performed under a microscope (Zeiss, Germany).

### Immunohistochemistry test

The tumor tissues were fixed with 4% paraformaldehyde, embedded in paraffin and cut into 4 μm thick sections. Then the sections were dewaxed with dimethylbenzene and rehydrated with gradient ethanol (100, 95, 90, 80 and 70%), for 5 min each, followed by blocking with serum. The samples were incubated with primary antibodies for EGFR and PCNA (1:200) for three hours each at 37°C and then washed with PBS three times. All sections were incubated with the HRP-conjugated secondary IgG antibodies (1:10,000) for 30 min at 37°C. Subsequent to being washed three times with PBS, the samples were stained with 3, 3-diaminobenzidine, kept at room temperature with dark for 10 min and then stained with hematoxylin. The samples were then dehydrated using an ethanol gradient (70, 80, 90, 95 and 100%), then rinsed in xylene for 10 min twice. The sections were observed and images were captured under an optical microscope.

### Statistical analysis

Date were the mean ± SEM (n=3). * P<0.05, ** P<0.01 and *** P<0.001. A p-value of less than 0.05 indicates a statistical significance. Compared with the control. All statistical treatments were performed using the SPSS 17.0 software.

## References

[1] Torre LA, Siegel RL, Jemal A (2016). Lung cancer statistics. Adv Exp Med Biol.

[2] Torre LA, Bray F, Siegel RL, Ferlay J, Lortet-Tieulent J, Jemal A (2015). Global cancer statistics, 2012. CA Cancer J Clin.

[3] Chunhacha P, Chanvorachote P (2012). Roles of caveolin-1 on anoikis resistance in non small cell lung cancer. Int J Physiol Pathophysiol Pharmacol.

[4] Hirsch FR, Scagliotti GV, Mulshine JL, Kwon R, Curran WJ, Wu YL, Paz-Ares L (2017). Lung cancer: current therapies and new targeted treatments. Lancet.

[5] Gridelli C, Rossi A, Carbone DP, Guarize J, Karachaliou N, Mok T, Petrella F, Spaggiari L, Rosell R (2015). Non-small-cell lung cancer. Nat Rev Dis Primers.

[6] Langhammer S, Scheerer J (2017). Breaking the crosstalk of the cellular tumorigenic network: hypothesis for addressing resistances to targeted therapies in advanced NSCLC. Oncotarget.

[7] Arteaga C (2003). Targeting HER1/EGFR: a molecular approach to cancer therapy. Semin Oncol.

[8] Russo A, Franchina T, Ricciardi GR, Picone A, Ferraro G, Zanghi M, Toscano G, Giordano A, Adamo V (2015). A decade of EGFR inhibition in EGFR-mutated non small cell lung cancer (NSCLC): Old successes and future perspectives. Oncotarget.

[9] Lee JG, Wu R (2012). Combination erlotinib-cisplatin and Atg3-mediated autophagy in erlotinib resistant lung cancer. PLoS One.

[10] Wang YS, Wang YH, Xia HP, Zhou SW, Schmid-Bindert G, Zhou CC (2012). MicroRNA-214 regulates the acquired resistance to gefitinib via the PTEN/AKT pathway in EGFR-mutant cell lines. Asian Pac J Cancer Prev.

[11] Wilson C, Nicholes K, Bustos D, Lin E, Song Q, Stephan JP, Kirkpatrick DS, Settleman J (2014). Overcoming EMT-associated resistance to anti-cancer drugs via Src/FAK pathway inhibition. Oncotarget.

[12] Engelman JA, Zejnullahu K, Mitsudomi T, Song Y, Hyland C, Park JO, Lindeman N, Gale CM, Zhao X, Christensen J, Kosaka T, Holmes AJ, Rogers AM (2007). MET amplification leads to gefitinib resistance in lung cancer by activating ERBB3 signaling. Science.

[13] Maitah MY, Ali S, Ahmad A, Gadgeel S, Sarkar FH (2011). Up-regulation of sonic hedgehog contributes to TGF-beta1-induced epithelial to mesenchymal transition in NSCLC cells. PLoS One.

[14] Ercan D, Xu C, Yanagita M, Monast CS, Pratilas CA, Montero J, Butaney M, Shimamura T, Sholl L, Ivanova EV, Tadi M, Rogers A, Repellin C (2012). Reactivation of ERK signaling causes resistance to EGFR kinase inhibitors. Cancer Discov.

[15] Sequist LV, Waltman BA, Dias-Santagata D, Digumarthy S, Turke AB, Fidias P, Bergethon K, Shaw AT, Gettinger S, Cosper AK, Akhavanfard S, Heist RS, Temel J (2011). Genotypic and histologica evolution of lung cancers acquiring resistance to EGFR inhibitors. Sci Transl Med.

[16] Yu HA, Arcila ME, Rekhtman N, Sima CS, Zakowski MF, Pao W, Kris MG, Miller VA, Ladanyi M, Riely GJ (2013). Analysis of tumor specimens at the time of acquired resistance to EGFR-TKI therapy in 155 patients with EGFR-mutant lung cancers. Clin Cancer Res.

[17] Sitruk-Ware R, Spitz IM (2003). Pharmacological properties of mifepristone: toxicology and safety in animal and human studies. Contraception.

[18] Ho PC, Yu Ng EH, Tang OS (2002). Mifepristone: contraceptive and non-contraceptive uses. Curr Opin Obstet Gynecol.

[19] Gaddy VT, Barrett JT, Delk JN, Kallab AM, Porter AG, Schoenlein PV (2004). Mifepristone induces growth arrest, caspase activation, and apoptosis of estrogen receptor-expressing, antiestrogen-resistant breast cancer cells. Clin Cancer Res.

[20] Fiscella J, Bonfiglio T, Winters P, Eisinger SH, Fiscella K (2011). Distinguishing features of endometrial pathology after exposure to the progesterone receptor modulator mifepristone. Hum Pathol.

[21] Ligr M, Li Y, Logan SK, Taneja S, Melamed J, Lepor H, Garabedian MJ, Lee P (2012). Mifepristone inhibits GRbeta coupled prostate cancer cell proliferation. J Urol.

[22] Wempe SL, Gamarra-Luques CD, Telleria CM (2013). Synergistic lethality of mifepristone and LY294002 in ovarian cancer cells. Cancer Growth Metastasis.

[23] Wang HY, Chang YL, Cheng CC, Chao MW, Lin SI, Pan SL, Hsu CC, Liu TW, Cheng HC, Tseng CP, Liu SJ, Tsai HJ, Chang HY, Hsu JT (2016). Glucocorticoids may compromise the effect of gefitinib in non-small cell lung cancer. Oncotarget.

[24] Chen JZ, Wang JC, Gao Y, Zeng RJ, Jiang Z, Zhu YW, Shao JW, Jia L (2014). A novel UPLC/MS/MS method for rapid determination of metapristone in rat plasma, a new cancer metastasis chemopreventive agent derived from mifepristone (RU486). J Pharm Biomed Anal.

[25] Chen J, Wang J, Shao J, Gao Y, Xu J, Yu S, Liu Z, Jia L (2014). The unique pharmacological characteristics of mifepristone (RU486): from terminating pregnancy to preventing cancer metastasis. Med Res Rev.

[26] Wang J, Chen J, Zhu Y, Zheng N, Liu J, Xiao Y, Lu Y, Dong H, Xie J, Yu S, Shao J, Jia L (2016). tc and *in vivo* efficacy and safety evaluation of metapristone and mifepristone as cancer metastatic chemopreventive agents. Biomed Pharmacother.

[27] Yu S, Yan C, Yang X, He S, Liu J, Qin C, Huang C, Lu Y, Tian Z, Jia L (2016). Pharmacoproteomic analysis reveals that metapristone (RU486 metabolite) intervenes E-cadherin and vimentin to realize cancer metastasis chemoprevention. Sci Rep.

[28] Zheng N, Chen J, Li T, Liu W, Liu J, Chen H, Wang J, Jia L (2017). Abortifacient metapristone (RU486 derivative) interrupts CXCL12/CXCR4 axis for ovarian metastatic chemoprevention. Mol Carcinog.

[29] Zheng G, Shen Z, Chen H, Liu J, Jiang K, Fan L, Jia L, Shao J (2017). Metapristone suppresses non-small cell lung cancer proliferation and metastasis via modulating RAS/RAF/MEK/MAPK signaling pathway. Biomed Pharmacother.

[30] da Cunha Santos G, Liu N, Tsao MS, Kamel-Reid S, Chin K, Geddie WR (2010). Detection of EGFR and KRAS mutations in fine-needle aspirates stored on Whatman FTA cards: is this the tool for biobanking cytological samples in the molecular era?. Cancer Cytopathol.

[31] Martinelli E, Troiani T, D'Aiuto E, Morgillo F, Vitagliano D, Capasso A, Costantino S, Ciuffreda LP, Merolla F, Vecchione L, De Vriendt V, Tejpar S, Nappi A (2013). Antitumor activity of pimasertib, a selective MEK 1/2 inhibitor, in combination with PI3K/mTOR inhibitors or with multi-targeted kinase inhibitors in pimasertib-resistant human lung and colorectal cancer cells. Int J Cancer.

[32] Fesik SW (2005). Promoting apoptosis as a strategy for cancer drug discovery. Nat Rev Cancer.

[33] Yarden Y, Pines G (2012). The ERBB network: at last, cancer therapy meets systems biology. Nat Rev Cancer.

[34] Wheeler DL, Dunn EF, Harari PM (2010). Understanding resistance to EGFR inhibitors-impact on future treatment strategies. Nat Rev Clin Oncol.

[35] Yarden Y, Sliwkowski MX (2001). Untangling the ErbB signalling network. Nat Rev Mol Cell Biol.

[36] Cross DA, Ashton SE, Ghiorghiu S, Eberlein C, Nebhan CA, Spitzler PJ, Orme JP, Finlay MR, Ward RA, Mellor MJ, Hughes G, Rahi A, Jacobs VN (2014). AZD9291, an irreversible EGFR TKI, overcomes T790M-mediated resistance to EGFR inhibitors in lung cancer. Cancer Discov.

[37] Tang ZH, Jiang XM, Guo X, Fong CM, Chen X, Lu JJ (2016). Characterization of osimertinib (AZD9291)-resistant non-small cell lung cancer NCI-H1975/OSIR cell line. Oncotarget.

[38] Wang J, Chen J, Wan L, Shao J, Lu Y, Zhu Y, Ou M, Yu S, Chen H, Jia L (2014). Synthesis, spectral characterization, and *in vitro* cellular activities of metapristone, a potential cancer metastatic chemopreventive agent derived from mifepristone (RU486). AAPS J.

[39] Evan GI, Vousden KH (2001). Proliferation, cell cycle and apoptosis in cancer. Nature.

[40] McIlwain DR, Berger T, Mak TW (2015). Caspase functions in cell death and disease. Cold Spring Harb Perspect Biol.

[41] Dent P (2014). Crosstalk between ERK, AKT, and cell survival. Cancer Biol Ther.

[42] Seshacharyulu P, Ponnusamy MP, Haridas D, Jain M, Ganti AK, Batra SK (2012). Targeting the EGFR signaling pathway in cancer therapy. Expert Opin Ther Targets.

[43] Nakamura H, Kurokawa J, Bai CX, Asada K, Xu J, Oren RV, Zhu ZI, Clancy CE, Isobe M, Furukawa T (2007). Progesterone regulates cardiac repolarization through a nongenomic pathway: an *in vitro* patch-clamp and computational modeling study. Circulation.

[44] Chen Y, Wang Y, Zhuang Y, Zhou F, Huang L (2012). Mifepristone increases the cytotoxicity of uterine natural killer cells by acting as a glucocorticoid antagonist via ERK activation. PLoS One.

[45] Shao JW, Dai YC, Xue JP, Wang JC, Lin FP, Guo YH (2011). *In vitro* and *in vivo* anticancer activity evaluation of ursolic acid derivatives. Eur J Med Chem.

[46] Dong H, Yang X, Xie J, Xiang L, Li Y, Ou M, Chi T, Liu Z, Yu S, Gao Y, Chen J, Shao J, Jia L (2015). UP12, a novel ursolic acid derivative with potential for targeting multiple signaling pathways in hepatocellular carcinoma. Biochem Pharmacol.

[47] Yang X, Li Y, Jiang W, Ou M, Chen Y, Xu Y, Wu Q, Zheng Q, Wu F, Wang L, Zou W, Zhang YJ, Shao J (2015). Synthesis and biological evaluation of novel ursolic acid derivatives as potential anticancer prodrugs. Chem Biol Drug Des.

[48] Shao J, Dai Y, Zhao W, Xie J, Xue J, Ye J, Jia L (2013). Intracellular distribution and mechanisms of actions of photosensitizer Zinc(II)-phthalocyanine solubilized in Cremophor EL against human hepatocellular carcinoma HepG2 cells. Cancer Lett.

[49] Xiang L, Chi T, Tang Q, Yang X, Ou M, Chen X, Yu X, Chen J, Ho RJ, Shao J, Jia L (2015). A pentacyclic triterpene natural product, ursolic acid and its prodrug US597 inhibit targets within cell adhesion pathway and prevent cancer metastasis. Oncotarget.

